# Notification that new names of prokaryotes, new combinations and new taxonomic opinions have appeared in volume 75, part 5 of the IJSEM

**DOI:** 10.1099/ijsem.0.006824

**Published:** 2025-09-01

**Authors:** Aharon Oren, Markus Göker

**Affiliations:** 1 The Institute of Life Sciences, The Hebrew University of Jerusalem, The Edmond J. Safra Campus, 9190401 Jerusalem, Israel; 2 Leibniz Institute DSMZ – German Collection of Microorganisms and Cell Cultures, Inhoffenstrasse 7B, 38124 Braunschweig, Germany

This listing of names of prokaryotes published in a previous issue of the *IJSEM* is provided as a service to microbiology to assist in the recognition of new names and new combinations. This procedure was proposed by the Judicial Commission [1]. The names given herein are listed according to the Rules of priority (i.e. time and date of publication of the articles on the journal’s platform and the order of valid publication of names in the original articles) [2].

**Table:** 

Order of article publication	Name/authors	Proposed as	Article no.	Page no.
1	*Janthinobacterium aestuarii* Pavloudi *et al*. 2025	sp. nov.	006768	9
2	*Guptibacillus* Bello *et al*. 2025	gen. nov.	006757	14
2	*Guptibacillus hwajinpoensis* (Yoon *et al*. 2004) Bello *et al*. 2025	comb. nov. [basonym: *Bacillus hwajinpoensis* Yoon *et al*. 2004]	006757	14
2	*Guptibacillus sedimenti* (Liu *et al*. 2023) Bello *et al*. 2025	comb. nov. [basonym: *Pseudalkalibacillus sedimenti* Liu *et al*. 2023]	006757	14
2	*Guptibacillus spartinae* (Liu *et al*. 2023) Bello *et al*. 2025	comb. nov. [basonym: *Pseudalkalibacillus spartinae* Liu *et al*. 2023]	006757	14
2	*Guptibacillus algicola* (Ivanova *et al*. 2004) Bello *et al*. 2025	comb. nov. [basonym: *Bacillus algicola* Ivanova *et al*. 2004]	006757	14
2	*Guptibacillus hemicentroti* (Chen *et al*. 2011) Bello *et al*. 2025	comb. nov. [basonym: *Bacillus hemicentroti* Chen *et al*. 2011]	006757	15
2	*Guptibacillaceae* Bello *et al*. 2025	fam. nov.	006757	15
2	*Exobacillus* Bello *et al*. 2025	gen. nov.	006757	15
2	*Exobacillus caeni* (Mo *et al*. 2020) Bello *et al*. 2025	comb. nov. [basonym: *Bacillus caeni* Mo *et al*. 2020]	006757	15
3	*Roseomonas xinghualingensis* Kang *et al*. 2025	sp. nov.	006775	6
4	*Labrys sedimenti* Csépányi *et al*. 2025	sp. nov.	006778	7
5	*Microaceticoccus* Cheng *et al*. 2025	gen. nov.	006773	8
5	*Microaceticoccus formicicus* Cheng *et al*. 2025	sp. nov.	006773	8
6	*Acetobacterium malicum* subsp. *malicum* (Tanaka and Pfennig 1990) Spring *et al*. 2025	comb. nov. [basonym: *Acetobacterium malicum* Tanaka and Pfennig 1990]	006783	8
6	*Acetobacterium malicum* subsp. *dehalogenans* Spring *et al*. 2025	subsp. nov.	006783	9
7	*Streptococcus hepaticus* Kirchner *et al*. 2025	sp. nov.	006776	4
8	*Streptomyces haneummycinicus* Uemura *et al*. 2025	sp. nov.	006777	5
9	*Streptomyces lycopersici* Qi *et al*. 2025	sp. nov.	006785	7
9	*Streptomyces cucumeris* Qi *et al*. 2025	sp. nov.	006785	8
10	*Neomoorella* Gtari and Ventura 2025	gen. nov.	006779	2
10	*Neomoorella thermoacetica* (Fontaine *et al*. 1942) Gtari and Ventura 2025	comb. nov. [basonym: *Clostridium thermoaceticum* Fontaine *et al*. 1942 (Approved Lists 1980)]	006779	2
10	*Neomoorella caeni* (Vecchini Santaella *et al*. 2023) Gtari and Ventura 2025	comb. nov. [illegitimate basonym: *Moorella caeni* Vecchini Santaella *et al*. 2023]	006779	2
10	*Neomoorella glycerini* (Slobodkin *et al*. 1997) Gtari and Ventura 2025	comb. nov. [illegitimate basonym: *Moorella glycerini* Slobodkin *et al*. 1997]	006779	2
10	*Neomoorella humiferrea* (Nepomnyashchaya *et al*. 2012) Gtari and Ventura 2025	comb. nov. [illegitimate basonym: *Moorella humiferrea* Nepomnyashchaya *et al*. 2012]	006779	2
10	*Neomoorella mulderi* (Balk *et al*. 2005) Gtari and Ventura 2025	comb. nov. [illegitimate basonym: *Moorella mulderi* Balk *et al*. 2005]	006779	3
10	*Neomoorella stamsii* (Alves *et al*. 2013) Gtari and Ventura 2025	comb. nov. [illegitimate basonym: *Moorella stamsii* Alves *et al*. 2013]	006779	3
10	*Neomoorella sulfitireducens* (Slobodkina *et al*. 2023) Gtari and Ventura 2025	comb. nov. [illegitimate basonym: *Moorella sulfitireducens* Slobodkina *et al*. 2023]	006779	3
10	*Neomoorella carbonis* Gtari and Ventura 2025*	sp. nov.	006779	3
10	*Neomoorellaceae* Gtari and Ventura 2025†	fam. nov.	006779	4
10	*Neomoorellales* Gtari and Ventura 2025‡	ord. nov.	006779	4
11	*Listeria tempestatis* Brown *et al*. 2025	sp. nov.	006774	6
11	*Listeria rocourtiae* subsp. *rocourtiae* (Leclercq *et al*. 2010) Brown *et al*. 2025	comb. nov. [basonym: *Listeria rocourtiae* Leclercq *et al*. 2010]	006774	7
11	*Listeria rocourtiae* subsp. *hofi* Brown *et al*. 2025	subsp. nov.	006774	7
12	*Imbroritus* Peeters *et al*. 2025§	gen. nov.	006781	7
12	*Imbroritus primus* Peeters *et al*. 2025	sp. nov.	006781	7
13	*Kitasatospora hibisci* Xiao *et al*. 2025	sp. nov.	006787	7
14	*Aquisediminimonadaceae* Wang *et al*. 2025	fam. nov.	006769	17
14	*Blastomonadaceae* Wang *et al*. 2025	fam. nov.	006769	17
14	*Parasphingopyxidaceae* Wang *et al*. 2025	fam. nov.	006769	17
14	*Pedomonadaceae* Wang *et al*. 2025	fam. nov.	006769	17
14	*Rhizorhabdaceae* Wang *et al*. 2025	fam. nov.	006769	17
14	*Sphingobiaceae* Wang *et al*. 2025	fam. nov.	006769	18
14	*Sphingomicrobiaceae* Wang *et al*. 2025	fam. nov.	006769	18
14	*Sphingopyxidaceae* Wang *et al*. 2025	fam. nov.	006769	18
14	*Sphingorhabdaceae* Wang *et al*. 2025	fam. nov.	006769	18
14	*Aestuarierythrobacter* Wang *et al*. 2025	gen. nov.	006769	18
14	*Aestuarierythrobacter aquiaggeris* (Jung *et al*. 2017) Wang *et al*. 2025	comb. nov. [basonym: *Altererythrobacter aquiaggeris* Jung *et al*. 2017]	006769	19
14	*Aestuarierythrobacter confluentis* (Park *et al*. 2016) Wang *et al*. 2025	comb. nov. [basonym: *Altererythrobacter confluentis* Park *et al*. 2016]	006769	19
14	*Aestuarierythrobacter sediminis* (Kim *et al*. 2016) Wang *et al*. 2025	comb. nov. [basonym: *Altererythrobacter sediminis* Kim *et al*. 2016]	006769	19
14	*Agrinovosphingobium* Wang *et al*. 2025	gen. nov.	006769	19
14	*Agrinovosphingobium ipomoeae* (Chen *et al*. 2017) Wang *et al*. 2025	comb. nov. [basonym: *Novosphingobium ipomoeae* Chen *et al*. 2017]	006769	19
14	*Allosphingosinicella deserti* (Liu *et al*. 2019) Wang *et al*. 2025	comb. nov. [basonym: *Sphingomonas deserti* Liu *et al*. 2019]	006769	20
14	*Allosphingosinicella flava* (Chhetri *et al*. 2021) Wang *et al*. 2025	comb. nov. [basonym: *Sphingosinicella flava* Chhetri *et al*. 2021]	006769	20
14	*Allosphingosinicella ginsenosidimutans* (Kim *et al*. 2019) Wang *et al*. 2025	comb. nov. [basonym: *Sphingosinicella ginsenosidimutans* Kim *et al*. 2019]	006769	20
14	*Allosphingosinicella humi* (Qiao *et al*. 2019) Wang *et al*. 2025	comb. nov. [basonym: *Sphingosinicella humi* Qiao *et al*. 2019]	006769	20
14	*Alteraquisediminimonas* Wang *et al*. 2025	gen. nov.	006769	20
14	*Alteraquisediminimonas sediminicola* (Jin *et al*. 2019) Wang *et al*. 2025	comb. nov. [basonym: *Aquisediminimonas sediminicola* Jin *et al*. 2019]	006769	20
14	*Alteraurantiacibacter lauratis* (Yuan *et al*. 2017) Wang *et al*. 2025	comb. nov. [basonym: *Altererythrobacter lauratis* Yuan *et al*. 2017]	006769	21
14	*Alteraurantiacibacter palmitatis* (Yuan *et al*. 2017) Wang *et al*. 2025	comb. nov. [basonym: *Altererythrobacter palmitatis* Yuan *et al*. 2017]	006769	21
14	*Alterinovosphingobium* Wang *et al*. 2025	gen. nov.	006769	21
14	*Alterinovosphingobium aerophilum* (Liu *et al*. 2021) Wang *et al*. 2025	comb. nov. [basonym: *Novosphingobium aerophilum* Liu *et al*. 2021]	006769	21
14	*Alterinovosphingobium bradum* (Sheu *et al*. 2016) Wang *et al*. 2025	comb. nov. [basonym: *Novosphingobium bradum* Sheu *et al*. 2016]	006769	21
14	*Alterinovosphingobium flavum* (Nguyen *et al*. 2016) Wang *et al*. 2025	comb. nov. [basonym: *Novosphingobium flavum* Nguyen *et al*. 2016]	006769	22
14	*Alterinovosphingobium humi* (Hyeon *et al*. 2017) Wang *et al*. 2025	comb. nov. [basonym: *Novosphingobium humi* Hyeon *et al*. 2017]	006769	22
14	*Alterinovosphingobium ovatum* (Chen *et al*. 2020) Wang *et al*. 2025	comb. nov. [basonym: *Novosphingobium ovatum* Chen *et al*. 2020]	006769	22
14	*Alterinovosphingobium piscinae* (Sheu *et al*. 2016) Wang *et al*. 2025	comb. nov. [basonym: *Novosphingobium piscinae* Sheu *et al*. 2016]	006769	22
14	*Alterinovosphingobium rosa* (Takeuchi *et al*. 1995) Wang *et al*. 2025	comb. nov. [basonym: *Sphingomonas rosa* Takeuchi *et al*. 1995]	006769	22
14	*Alterinovosphingobium umbonatum* (Sheu *et al*. 2020) Wang *et al*. 2025	comb. nov. [basonym: *Novosphingobium umbonatum* Sheu *et al*. 2020]	006769	23
14	*Alterirhizorhabdus* Wang *et al*. 2025	gen. nov.	006769	23
14	*Alterirhizorhabdus profundi* (Yang *et al*. 2020) Wang *et al*. 2025	comb. nov. [basonym: *Sphingomonas profundi* Yang *et al*. 2020]	006769	23
14	*Alterirhizorhabdus solaris* (Tanner *et al*. 2020) Wang *et al*. 2025	comb. nov. [basonym: *Sphingomonas solaris* Tanner *et al*. 2020]	006769	23
14	*Alterisphingobium* Wang *et al*. 2025	gen. nov.	006769	23
14	*Alterisphingobium fluviale* (Sheu *et al*. 2020) Wang *et al*. 2025	comb. nov. [basonym: *Sphingobium fluviale* Sheu *et al*. 2020]	006769	23
14	*Alterisphingobium jiangsuense* (Zhang *et al*. 2012) Wang *et al*. 2025	comb. nov. [basonym: *Sphingobium jiangsuense* Zhang *et al*. 2012]	006769	24
14	*Alterisphingobium subterraneum* (Lee *et al*. 2015) Wang *et al*. 2025	comb. nov. [basonym: *Sphingobium subterraneum* Lee *et al*. 2015]	006769	24
14	*Alterisphingomonas* Wang *et al*. 2025	gen. nov.	006769	24
14	*Alterisphingomonas asaccharolytica* (Takeuchi *et al*. 1995) Wang *et al*. 2025	comb. nov. [basonym: *Sphingomonas asaccharolytica* Takeuchi *et al*. 1995]	006769	24
14	*Alterisphingomonas mali* (Takeuchi *et al*. 1995) Wang *et al*. 2025	comb. nov. [basonym: *Sphingomonas mali* Takeuchi *et al*. 1995]	006769	24
14	*Alterisphingomonas panacisoli* (Maeng *et al*. 2021) Wang *et al*. 2025	comb. nov. [basonym: *Sphingomonas panacisoli* Maeng *et al*. 2021]	006769	25
14	*Alterisphingomonas pruni* (Takeuchi *et al*. 1995) Wang *et al*. 2025	comb. nov. [basonym: *Sphingomonas pruni* Takeuchi *et al*. 1995]	006769	25
14	*Alterisphingomonas radiodurans* (Liu *et al*. 2022) Wang *et al*. 2025	comb. nov. [basonym: *Sphingomonas radiodurans* Liu *et al*. 2022]	006769	25
14	*Alterisphingopyxis* Wang *et al*. 2025	gen. nov.	006769	25
14	*Alterisphingopyxis lacunae* (Sheu *et al*. 2020) Wang *et al*. 2025	comb. nov. [basonym: *Sphingomonas lacunae* Sheu *et al*. 2020]	006769	25
14	*Alterisphingorhabdus* Wang *et al*. 2025	gen. nov.	006769	26
14	*Alterisphingorhabdus lutea* (Baek *et al*. 2019) Wang *et al*. 2025	comb. nov. [basonym: *Sphingorhabdus lutea* Baek *et al*. 2019]	006769	26
14	*Alteristakelama* Wang *et al*. 2025	gen. nov.	006769	26
14	*Alteristakelama azotifigens* (Xie and Yokota 2006) Wang *et al*. 2025	comb. nov. [basonym: *Sphingomonas azotifigens* Xie and Yokota 2006]	006769	26
14	*Alteristakelama canadensis* (Abraham *et al*. 2013) Wang *et al*. 2025	comb. nov. [basonym: *Sphingomonas canadensis* Abraham *et al*. 2013]	006769	26
14	*Alteristakelama gei* (Zhu *et al*. 2015) Wang *et al*. 2025	comb. nov. [basonym: *Sphingomonas gei* Zhu *et al*. 2015]	006769	26
14	*Alteristakelama hengshuiensis* (Wei *et al*. 2015) Wang *et al*. 2025	comb. nov. [basonym: *Sphingomonas hengshuiensis* Wei *et al*. 2015]	006769	27
14	*Alteristakelama koreensis* (Lee *et al*. 2001) Wang *et al*. 2025	comb. nov. [basonym: *Sphingomonas koreensis* Lee *et al*. 2001]	006769	27
14	*Alteristakelama kyeonggiensis* (Son *et al*. 2014) Wang *et al*. 2025	comb. nov. [basonym: *Sphingomonas kyeonggiensis* corrig. Son *et al*. 2014]	006769	27
14	*Alteristakelama leidyi* (Poindexter 1964) Wang *et al*. 2025	comb. nov. [basonym: *Caulobacter leidyi* corrig. Poindexter 1964 (Approved Lists 1980)]	006769	27
14	*Alteristakelama naasensis* (Kim *et al*. 2014) Wang *et al*. 2025	comb. nov. [basonym: *Sphingomonas naasensis* Kim *et al*. 2014]	006769	27
14	*Alteristakelama pituitosa* (Denner *et al*. 2001) Wang *et al*. 2025	comb. nov. [basonym: *Sphingomonas pituitosa* Denner *et al*. 2001]	006769	28
14	*Alteristakelama pokkalii* (Menon *et al*. 2021) Wang *et al*. 2025	comb. nov. [basonym: *Sphingomonas pokkalii* Menon *et al*. 2021]	006769	28
14	*Alteristakelama psychrotolerans* (Luo *et al*. 2022) Wang *et al*. 2025	comb. nov. [basonym: *Sphingomonas psychrotolerans* Luo *et al*. 2022]	006769	28
14	*Alteristakelama soli* (Yang *et al*. 2006) Wang *et al*. 2025	comb. nov. [basonym: *Sphingomonas soli* Yang *et al*. 2006]	006769	28
14	*Alteristakelama suaedae* (Zhang *et al*. 2020) Wang *et al*. 2025	comb. nov. [basonym: *Sphingomonas suaedae* Zhang *et al*. 2020]	006769	28
14	*Alteristakelama trueperi* (Kämpfer *et al*. 1997) Wang *et al*. 2025	comb. nov. [basonym: *Sphingomonas trueperi* Kämpfer *et al*. 1997]	006769	28
14	*Alteristakelama turrisvirgatae* (Thaller *et al*. 2018) Wang *et al*. 2025	comb. nov. [basonym: *Sphingomonas turrisvirgatae* Thaller *et al*. 2018]	006769	29
14	*Alteristakelama xinjiangensis* (An *et al*. 2011) Wang *et al*. 2025	comb. nov. [basonym: *Sphingomonas xinjiangensis* An *et al*. 2011]	006769	29
14	*Aquisediminimonas godavariana* (Jani *et al*. 2019) Wang *et al*. 2025	comb. nov. [basonym: *Chakrabartia godavariana* Jani *et al*. 2019]	006769	29
14	*Caenibius estronivorus* (Qin *et al*. 2021) Wang *et al*. 2025	comb. nov. [basonym: *Altererythrobacter estronivorus* Qin *et al*. 2021]	006769	29
14	*Caenibius fulvus* (Dahal and Kim 2018) Wang *et al*. 2025	comb. nov. [basonym: *Altererythrobacter fulvus* Dahal and Kim 2018]	006769	29
14	*Edaphosphingomonas* Wang *et al*. 2025	gen. nov.	006769	30
14	*Edaphosphingomonas fennica* (Wittich *et al*. 2007) Wang *et al*. 2025	comb. nov. [basonym: *Sphingomonas fennica* Wittich *et al*. 2007]	006769	30
14	*Edaphosphingomonas haloaromaticamans* (Wittich *et al*. 2007) Wang *et al*. 2025	comb. nov. [basonym: *Sphingomonas haloaromaticamans* Wittich *et al*. 2007]	006769	30
14	*Edaphosphingomonas laterariae* (Kaur *et al*. 2012) Wang *et al*. 2025	comb. nov. [basonym: *Sphingomonas laterariae* Kaur *et al*. 2012]	006769	30
14	*Flavinovosphingobium* Wang *et al*. 2025	gen. nov.	006769	30
14	*Flavinovosphingobium sediminicola* (Baek *et al*. 2011) Wang *et al*. 2025	comb. nov. [basonym: *Novosphingobium sediminicola* Baek *et al*. 2011]	006769	31
14	*Flavisphingomonas* Wang *et al*. 2025	gen. nov.	006769	31
14	*Flavisphingomonas formosensis* (Lin *et al*. 2012) Wang *et al*. 2025	comb. nov. [basonym: *Sphingomonas formosensis* Lin *et al*. 2012]	006769	31
14	*Flavisphingopyxis* Wang *et al*. 2025	gen. nov.	006769	31
14	*Flavisphingopyxis soli* (Liu *et al*. 2020) Wang *et al*. 2025	comb. nov. [basonym: *Sphingorhabdus soli* Liu *et al*. 2020]	006769	31
14	*Glacieibacterium algoris* (Tahon *et al*. 2022) Wang *et al*. 2025	comb. nov. [basonym: *Chioneia algoris* Tahon *et al*. 2022]	006769	31
14	*Glacieibacterium arshaanense* (Phurbu *et al*. 2020) Wang *et al*. 2025	comb. nov. [basonym: *Polymorphobacter arshaanensis* Phurbu *et al*. 2020]	006769	32
14	*Glacieibacterium brumae* (Tahon *et al*. 2022) Wang *et al*. 2025	comb. nov. [basonym: *Chioneia brumae* Tahon *et al*. 2022]	006769	32
14	*Glacieibacterium frigoris* Wang *et al*. 2025	nom. nov. [basonym: *Chioneia frigida* Tahon *et al*. 2022]	006769	32
14	*Glacieibacterium hiemis* (Tahon *et al*. 2022) Wang *et al*. 2025	comb. nov. [basonym: *Chioneia hiemis* Tahon *et al*. 2022]	006769	32
14	*Glacieibacterium megasporae* (Noh *et al*. 2022) Wang *et al*. 2025	comb. nov. [basonym: *Polymorphobacter megasporae* Noh *et al*. 2022]	006769	32
14	*Humisphingomonas* Wang *et al*. 2025	gen. nov.	006769	32
14	*Humisphingomonas gilva* (Zhu *et al*. 2019) Wang *et al*. 2025	comb. nov. [basonym: *Sphingomonas gilva* Zhu *et al*. 2019]	006769	33
14	*Neorhizorhabdus* Wang *et al*. 2025	gen. nov.	006769	33
14	*Neorhizorhabdus crusticola* (Zhang *et al*. 2017) Wang *et al*. 2025	comb. nov. [basonym: *Sphingomonas crusticola* Zhang *et al*. 2017]	006769	33
14	*Neorhizorhabdus naphthae* (Chaudhary and Kim 2016) Wang *et al*. 2025	comb. nov. [basonym: *Sphingomonas naphthae* Chaudhary and Kim 2016]	006769	33
14	*Neorhizorhabdus oleivorans* (Chen *et al*. 2018) Wang *et al*. 2025	comb. nov. [basonym: *Sphingomonas oleivorans* Chen *et al*. 2018]	006769	33
14	*Neorhizorhabdus vulcanisoli* (Lee *et al*. 2015) Wang *et al*. 2025	comb. nov. [basonym: *Sphingomonas vulcanisoli* Lee *et al*. 2015]	006769	34
14	*Neosandaracinobacteroides* Wang *et al*. 2025	gen. nov.	006769	34
14	*Neosandaracinobacteroides saxicola* (Tang *et al*. 2023) Wang *et al*. 2025	comb. nov. [basonym: *Sandaracinobacteroides saxicola* Tang *et al*. 2023]	006769	34
14	*Novistakelama* Wang *et al*. 2025	gen. nov.	006769	34
14	*Novistakelama desiccabilis* (Reddy and Garcia-Pichel 2007) Wang *et al*. 2025	comb. nov. [basonym: *Sphingomonas desiccabilis* Reddy and Garcia-Pichel 2007]	006769	35
14	*Novistakelama hankookensis* (Yoon *et al*. 2009) Wang *et al*. 2025	comb. nov. [basonym: *Sphingomonas hankookensis* Yoon *et al*. 2009]	006769	35
14	*Novistakelama panni* (Busse *et al*. 2005) Wang *et al*. 2025	comb. nov. [basonym: *Sphingomonas panni* Busse *et al*. 2005]	006769	35
14	*Paranovosphingobium* Wang *et al*. 2025	gen. nov.	006769	35
14	*Paranovosphingobium aquiterrae* (Lee *et al*. 2014) Wang *et al*. 2025	comb. nov. [basonym: *Novosphingobium aquiterrae* Lee *et al*. 2014]	006769	35
14	*Paranovosphingobium arvoryzae* (Sheu *et al*. 2018) Wang *et al*. 2025	comb. nov. [basonym: *Novosphingobium arvoryzae* Sheu *et al*. 2018]	006769	36
14	*Paranovosphingobium kunmingense* (Xie *et al*. 2014) Wang *et al*. 2025	comb. nov. [basonym: *Novosphingobium kunmingense* Xie *et al*. 2014]	006769	36
14	*Paraquisediminimonas* Wang *et al*. 2025	gen. nov.	006769	36
14	*Paraquisediminimonas paeninsulae* (Geng *et al*. 2019) Wang *et al*. 2025	comb. nov. [basonym: *Sphingomonas paeninsulae* Geng *et al*. 2019]	006769	36
14	*Pararhizorhabdus* Wang *et al*. 2025	gen. nov.	006769	36
14	*Pararhizorhabdus arantia* (Jia *et al*. 2016) Wang *et al*. 2025	comb. nov. [basonym: *Sphingomonas arantia* Jia *et al*. 2016]	006769	37
14	*Pararhizorhabdus jatrophae* (Madhaiyan *et al*. 2017) Wang *et al*. 2025	comb. nov. [basonym: *Sphingomonas jatrophae* Madhaiyan *et al*. 2017]	006769	37
14	*Pararhizorhabdus montana* (Manandhar *et al*. 2018) Wang *et al*. 2025	comb. nov. [basonym: *Sphingomonas montana* Manandhar *et al*. 2018]	006769	37
14	*Pararhizorhabdus prati* (Manandhar *et al*. 2016) Wang *et al*. 2025	comb. nov. [basonym: *Sphingomonas prati* Manandhar *et al*. 2016]	006769	37
14	*Parasphingobium* Wang *et al*. 2025	gen. nov.	006769	37
14	*Parasphingobium lignivorans* (Allemann *et al*. 2023) Wang *et al*. 2025	comb. nov. [basonym: *Sphingobium lignivorans* Allemann *et al*. 2023]	006769	38
14	*Parasphingobium sufflavum* (Sheu *et al*. 2013) Wang *et al*. 2025	comb. nov. [basonym: *Sphingobium sufflavum* Sheu *et al*. 2013]	006769	38
14	*Parasphingobium xanthum* (Francis *et al*. 2014) Wang *et al*. 2025	comb. nov. [basonym: *Sphingobium xanthum* Francis *et al*. 2014]	006769	38
14	*Parasphingomonas* Wang *et al*. 2025	gen. nov.	006769	38
14	*Parasphingomonas aliaeris* (Heidler von Heilborn *et al*. 2021) Wang *et al*. 2025	comb. nov. [basonym: *Sphingomonas aliaeris* Heidler von Heilborn *et al*. 2021]	006769	38
14	*Parasphingomonas alpina* (Margesin *et al*. 2012) Wang *et al*. 2025	comb. nov. [basonym: *Sphingomonas alpina* Margesin *et al*. 2012]	006769	39
14	*Parasphingomonas aquatica* (Choi *et al*. 2017) Wang *et al*. 2025	comb. nov. [basonym: *Sphingomonas aquatica* Choi *et al*. 2017]	006769	39
14	*Parasphingomonas aracearum* (Wang *et al*. 2019) Wang *et al*. 2025	comb. nov. [basonym: *Sphingomonas aracearum* Wang *et al*. 2019]	006769	39
14	*Parasphingomonas echinoides* (Heumann 1962) Wang *et al*. 2025	comb. nov. [basonym: *Pseudomonas echinoides* Heumann 1962 (Approved Lists 1980)]	006769	39
14	*Parasphingomonas glacialis* (Zhang *et al*. 2011) Wang *et al*. 2025	comb. nov. [basonym: *Sphingomonas glacialis* Zhang *et al*. 2011]	006769	39
14	*Parasphingomonas hylomeconis* (Akbar *et al*. 2015) Wang *et al*. 2025	comb. nov. [basonym: *Sphingomonas hylomeconis* Akbar *et al*. 2015]	006769	40
14	*Parasphingomonas panacis* (Singh e*t al*. 2017) Wang *et al*. 2025	comb. nov. [basonym: *Sphingomonas panacis* Singh e*t al*. 2017]	006769	40
14	*Parasphingomonas populi* (Li *et al*. 2020) Wang *et al*. 2025	comb. nov. [basonym: *Sphingomonas populi* Li *et al*. 2020]	006769	40
14	*Parasphingomonas psychrolutea* (Liu *et al*. 2015) Wang *et al*. 2025	comb. nov. [basonym: *Sphingomonas psychrolutea* Liu *et al*. 2015]	006769	40
14	*Parasphingomonas qilianensis* (Piao *et al*. 2016) Wang *et al*. 2025	comb. nov. [basonym: *Sphingomonas qilianensis* Piao *et al*. 2016]	006769	40
14	*Parastakelama* Wang *et al*. 2025	gen. nov.	006769	40
14	*Parastakelama aestuarii* (Roh *et al*. 2009) Wang *et al*. 2025	comb. nov. [basonym: *Sphingomonas aestuarii* Roh *et al*. 2009]	006769	41
14	*Parastakelama baiyangensis* (Wei *et al*. 2022) Wang *et al*. 2025	comb. nov. [basonym: *Sphingomonas baiyangensis* Wei *et al*. 2022]	006769	41
14	*Parastakelama japonica* (Romanenko *et al*. 2009) Wang *et al*. 2025	comb. nov. [basonym: *Sphingomonas japonica* Romanenko *et al*. 2009]	006769	41
14	*Parastakelama spermidinifaciens* (Feng *et al*. 2017) Wang *et al*. 2025	comb. nov. [basonym: *Sphingomonas spermidinifaciens* Feng *et al*. 2017]	006769	41
14	*Parastakelama yantingensis* (Huang *et al*. 2014) Wang *et al*. 2025	comb. nov. [basonym: *Sphingomonas yantingensis* Huang *et al*. 2014]	006769	41
14	*Pseudoblastomonas* Wang *et al*. 2025	gen. nov.	006769	42
14	*Pseudoblastomonas halimionae* (Fidalgo *et al*. 2017) Wang *et al*. 2025	comb. nov. [basonym: *Altererythrobacter halimionae* Fidalgo *et al*. 2017]	006769	42
14	*Pseudoblastomonas marina* (Meng *et al*. 2017) Wang *et al*. 2025	comb. nov. [basonym: *Blastomonas marina* Meng *et al*. 2017]	006769	42
14	*Pseudonovosphingobium* Wang *et al*. 2025	gen. nov.	006769	42
14	*Pseudonovosphingobium aquimarinum* (Le *et al*. 2020) Wang *et al*. 2025	comb. nov. [basonym: *Novosphingobium aquimarinum* Le *et al*. 2020]	006769	43
14	*Pseudonovosphingobium aureum* (Yoo *et al*. 2021) Wang *et al*. 2025	comb. nov. [basonym: *Novosphingobium aureum* Yoo *et al*. 2021]	006769	43
14	*Pseudonovosphingobium barchaimii* (Niharika *et al*. 2013) Wang *et al*. 2025	comb. nov. [basonym: *Novosphingobium barchaimii* Niharika *et al*. 2013]	006769	43
14	*Pseudonovosphingobium chloroacetimidivorans* (Chen *et al*. 2014) Wang *et al*. 2025	comb. nov. [basonym: *Novosphingobium chloroacetimidivorans* Chen *et al*. 2014]	006769	43
14	*Pseudonovosphingobium clariflavum* (Zhang *et al*. 2017) Wang *et al*. 2025	comb. nov. [basonym: *Novosphingobium clariflavum* Zhang *et al*. 2017]	006769	43
14	*Pseudonovosphingobium colocasiae* (Chen *et al*. 2016) Wang *et al*. 2025	comb. nov. [basonym: *Novosphingobium colocasiae* Chen *et al*. 2016]	006769	44
14	*Pseudonovosphingobium decolorationis* (Chen *et al*. 2021) Wang *et al*. 2025	comb. nov. [basonym: *Novosphingobium decolorationis* Chen *et al*. 2021]	006769	44
14	*Pseudonovosphingobium endophyticum* (Li *et al*. 2016) Wang *et al*. 2025	comb. nov. [basonym: *Novosphingobium endophyticum* Li *et al*. 2016]	006769	44
14	*Pseudonovosphingobium fluoreni* (Gao *et al*. 2015) Wang *et al*. 2025	comb. nov. [basonym: *Novosphingobium fluoreni* Gao *et al*. 2015]	006769	44
14	*Pseudonovosphingobium gossypii* (Kämpfer *et al*. 2015) Wang *et al*. 2025	comb. nov. [basonym: *Novosphingobium gossypii* Kämpfer *et al*. 2015]	006769	44
14	*Pseudonovosphingobium guangzhouense* (Sha *et al*. 2017) Wang *et al*. 2025	comb. nov. [basonym: *Novosphingobium guangzhouense* Sha *et al*. 2017]	006769	45
14	*Pseudonovosphingobium indicum* (Yuan *et al*. 2009) Wang *et al*. 2025	comb. nov. [basonym: *Novosphingobium indicum* Yuan *et al*. 2009]	006769	45
14	*Pseudonovosphingobium lindaniclasticum* (Saxena *et al*. 2013) Wang *et al*. 2025	comb. nov. [basonym: *Novosphingobium lindaniclasticum* Saxena *et al*. 2013]	006769	45
14	*Pseudonovosphingobium malaysiense* (Lee *et al*. 2014) Wang *et al*. 2025	comb. nov. [basonym: *Novosphingobium malaysiense* Lee *et al*. 2014]	006769	45
14	*Pseudonovosphingobium marinum* (Huo *et al*. 2015) Wang *et al*. 2025	comb. nov. [basonym: *Novosphingobium marinum* Huo *et al*. 2015]	006769	45
14	*Pseudonovosphingobium mathurense* (Gupta *et al*. 2009) Wang *et al*. 2025	comb. nov. [basonym: *Novosphingobium mathurense* Gupta *et al*. 2009]	006769	46
14	*Pseudonovosphingobium naphthalenivorans* (Suzuki and Hiraishi 2008) Wang *et al*. 2025	comb. nov. [basonym: *Novosphingobium naphthalenivorans* Suzuki and Hiraishi 2008]	006769	46
14	*Pseudonovosphingobium panipatense* (Gupta *et al*. 2009) Wang *et al*. 2025	comb. nov. [basonym: *Novosphingobium panipatense* Gupta *et al*. 2009]	006769	46
14	*Pseudonovosphingobium pentaromativorans* (Sohn *et al*. 2004) Wang *et al*. 2025	comb. nov. [basonym: *Novosphingobium pentaromativorans* Sohn *et al*. 2004]	006769	46
14	*Pseudonovosphingobium resinovorum* (Delaporte and Daste 1956) Wang *et al*. 2025	comb. nov. [basonym: *Flavobacterium resinovorum* Delaporte and Daste 1956 (Approved Lists 1980)]	006769	46
14	*Pseudonovosphingobium silvae* (Feng *et al*. 2020) Wang *et al*. 2025	comb. nov. [basonym: *Novosphingobium silvae* Feng *et al*. 2020]	006769	47
14	*Pseudonovosphingobium soli* (Kämpfer *et al*. 2011) Wang *et al*. 2025	comb. nov. [basonym: *Novosphingobium soli* Kämpfer *et al*. 2011]	006769	47
14	*Pseudopontixanthobacter muriae* (Azpiazu-Muniozguren *et al*. 2021) Wang *et al*. 2025	comb. nov. [basonym: *Altererythrobacter muriae* Azpiazu-Muniozguren *et al*. 2021]	006769	47
14	*Pseudosphingomonas* Wang *et al*. 2025	gen. nov.	006769	47
14	*Pseudosphingomonas agri* (Siddiqi *et al*. 2017) Wang *et al*. 2025	comb. nov. [basonym: *Sphingomonas agri* Siddiqi *et al*. 2017]	006769	47
14	*Pseudosphingomonas arenae* (Dong *et al*. 2022) Wang *et al*. 2025	comb. nov. [basonym: *Sphingomonas arenae* Dong *et al*. 2022]	006769	47
14	*Pseudosphingomonas astaxanthinifaciens* (Asker *et al*. 2008) Wang *et al*. 2025	comb. nov. [basonym: *Sphingomonas astaxanthinifaciens* Asker *et al*. 2008]	006769	48
14	*Pseudosphingomonas daechungensis* (Huy *et al*. 2014) Wang *et al*. 2025	comb. nov. [basonym: *Sphingomonas daechungensis* Huy *et al*. 2014]	006769	48
14	*Pseudosphingomonas edaphi* (Kim *et al*. 2020) Wang *et al*. 2025	comb. nov. [basonym: *Sphingomonas edaphi* Kim *et al*. 2020]	006769	48
14	*Pseudosphingomonas ginkgonis* (Cha *et al*. 2019) Wang *et al*. 2025	comb. nov. [basonym: *Sphingomonas ginkgonis* Cha *et al*. 2019]	006769	48
14	*Pseudosphingomonas ginsengisoli* (An *et al*. 2013) Wang *et al*. 2025	comb. nov. [basonym: *Sphingomonas ginsengisoli* An *et al*. 2013]	006769	48
14	*Pseudosphingomonas jaspsi* (Asker *et al*. 2007) Wang *et al*. 2025	comb. nov. [basonym: *Sphingomonas jaspsi* Asker *et al*. 2007]	006769	49
14	*Pseudosphingomonas kaistensis* (Kim *et al*. 2007) Wang *et al*. 2025	comb. nov. [basonym: *Sphingomonas kaistensis* Kim *et al*. 2007]	006769	49
14	*Pseudosphingomonas lutea* (Lee *et al*. 2016) Wang *et al*. 2025	comb. nov. [basonym: *Sphingomonas lutea* Lee *et al*. 2016]	006769	49
14	*Pseudosphingomonas mesophila* (Li *et al*. 2019) Wang *et al*. 2025	comb. nov. [basonym: *Sphingomonas mesophila* Li *et al*. 2019]	006769	49
14	*Pseudosphingomonas piscis* (Hyun *et al*. 2022) Wang *et al*. 2025	comb. nov. [basonym: *Sphingomonas piscis* Hyun *et al*. 2022]	006769	49
14	*Pseudosphingomonas rhizophila* (Yan *et al*. 2018) Wang *et al*. 2025	comb. nov. [basonym: *Sphingomonas rhizophila* Yan *et al*. 2018]	006769	50
14	*Pseudosphingomonas sabuli* (Kang *et al*. 2021) Wang *et al*. 2025	comb. nov. [basonym: *Sphingomonas sabuli* Kang *et al*. 2021]	006769	50
14	*Pseudosphingomonas segetis* (Lee and Whang 2020) Wang *et al*. 2025	comb. nov. [basonym: *Sphingomonas segetis* Lee and Whang 2020]	006769	50
14	*Pseudosphingomonas sinipercae* (Hyun *et al*. 2022) Wang *et al*. 2025	comb. nov. [basonym: *Sphingomonas sinipercae* Hyun *et al*. 2022]	006769	50
14	*Pseudostakelama* Wang *et al*. 2025	gen. nov.	006769	50
14	*Pseudostakelama cannabina* (Jeon *et al*. 2022) Wang *et al*. 2025	comb. nov. [basonym: *Sphingomonas cannabina* Jeon *et al*. 2022]	006769	50
14	*Pseudostakelama floccifaciens* (Fan *et al*. 2019) Wang *et al*. 2025	comb. nov. [basonym: *Sphingomonas floccifaciens* Fan *et al*. 2019]	006769	51
14	*Pseudostakelama guangdongensis* (Feng *et al*. 2014) Wang *et al*. 2025	comb. nov. [basonym: *Sphingomonas guangdongensis* Feng *et al*. 2014]	006769	51
14	*Qipengyuania atrilutea* (Xue *et al*. 2021) Wang *et al*. 2025	comb. nov. [basonym: *Actirhodobacter atriluteus* Xue *et al*. 2021]	006769	51
14	*Qipengyuania lutea* Wang *et al*. 2025	nom. nov. [basonym: *Altererythrobacter aurantiacus* Zhang *et al*. 2016]	006769	51
14	*Rhizorhabdus crocodyli* (Sheu *et al*. 2019) Wang *et al*. 2025	comb. nov. [basonym: *Sphingomonas crocodyli* Sheu *et al*. 2019]	006769	51
14	*Rhizorhabdus montanisoli* (Xu *et al*. 2020) Wang *et al*. 2025	comb. nov. [basonym: *Sphingomonas montanisoli* Xu *et al*. 2020]	006769	52
14	*Sandarakinorhabdus fusca* (Jia *et al*. 2015) Wang *et al*. 2025	comb. nov. [basonym: *Polymorphobacter fuscus* Jia *et al*. 2015]	006769	52
14	*Sandarakinorhabdus glacialis* (Xing *et al*. 2017) Wang *et al*. 2025	comb. nov. [basonym: *Polymorphobacter glacialis* Xing *et al*. 2017]	006769	52
14	*Solisphingomonas* Wang *et al*. 2025	gen. nov.	006769	52
14	*Solisphingomonas chungangi* (Akter and Huq 2020) Wang *et al*. 2025	comb. nov. [basonym: *Sphingomonas chungangi* Akter and Huq 2020]	006769	52
14	*Solisphingomonas morindae* (Liu *et al*. 2015) Wang *et al*. 2025	comb. nov. [basonym: *Sphingomonas morindae* Liu *et al*. 2015]	006769	52
14	*Solisphingomonas oligoaromativorans* (Han *et al*. 2014) Wang *et al*. 2025	comb. nov. [basonym: *Sphingomonas oligoaromativorans* Han *et al*. 2014]	006769	53
14	*Solisphingomonas quercus* (Shen *et al*. 2022) Wang *et al*. 2025	comb. nov. [basonym: *Sphingomonas quercus* Shen *et al*. 2022]	006769	53
14	*Stakelama portus* (Hetharua *et al*. 2019) Wang *et al*. 2025	comb. nov. [basonym: *Sphingosinithalassobacter portus* Hetharua *et al*. 2019]	006769	53
14	*Stakelama tenebrarum* (Zheng and Sun 2020) Wang *et al*. 2025	comb. nov. [basonym: *Sphingosinithalassobacter tenebrarum* Zheng and Sun 2020]	006769	53
14	*Yabuuchia* Wang *et al*. 2025	gen. nov.	006769	53
14	*Yabuuchia boeckii* (Chen *et al*. 2013) Wang *et al*. 2025	comb. nov. [basonym: *Sphingobium boeckii* Chen *et al*. 2013]	006769	54
14	*Yabuuchia cavernae* (Zhu *et al*. 2022) Wang *et al*. 2025	comb. nov. [basonym: *Sphingomonas cavernae* Zhu *et al*. 2022]	006769	54
14	*Yabuuchia colocasiae* (Lin *et al*. 2018) Wang *et al*. 2025	comb. nov. [basonym: *Sphingomonas colocasiae* Lin *et al*. 2018]	006769	54
14	*Alteriyabuuchia* Wang *et al*. 2025	gen. nov.	006769	54
14	*Alteriyabuuchia sanxanigenens* (Huang *et al*. 2009) Wang *et al*. 2025	comb. nov. [basonym: *Sphingomonas sanxanigenens* Huang *et al*. 2009]	006769	54
14	*Alteriyabuuchia zeicaulis* (Gao *et al*. 2016) Wang *et al*. 2025	comb. nov. [basonym: *Sphingomonas zeicaulis* Gao *et al*. 2016]	006769	55
14	*Parayabuuchia* Wang *et al*. 2025	gen. nov.	006769	55
14	*Parayabuuchia changbaiensis* (Zhang *et al*. 2010) Wang *et al*. 2025	comb. nov. [basonym: *Sphingomonas changbaiensis* Zhang *et al*. 2010]	006769	55
14	*Parayabuuchia flavalba* (Zhou *et al*. 2019) Wang *et al*. 2025	comb. nov. [basonym: *Sphingomonas flavalba* Zhou *et al*. 2019]	006769	55
14	*Parayabuuchia jejuensis* (Park *et al*. 2012) Wang *et al*. 2025	comb. nov. [basonym: *Sphingomonas jejuensis* Park *et al*. 2012]	006769	55
15	*Vreelandella arctica* Weng *et al*. 2025	sp. nov.	006791	6
15	*Vreelandella indica* Weng *et al*. 2025	sp. nov.	006791	9
16	*Nocardia sichangensis* Sripreechasak *et al*. 2025	sp. nov.	006784	7
17	*Humidisolicoccus* Kim and Kim 2025	gen. nov.	006786	5
17	*Humidisolicoccus flavus* Kim and Kim 2025	sp. nov.	006786	5
18	*Moraxella veridica* Nguyen *et al*. 2025	sp. nov.	006797	6
19	*Olsenella kribbiana* Bai *et al*. 2025	sp. nov.	006795	6
20	*Comamonas trifloxystrobinivorans* Zhu *et al*. 2025	sp. nov.	006796	9
21	*Streptomyces explomaris* Shu *et al*. 2025	sp. nov.	006711	8
22	*Catenibacterium mitsuokai* subsp. *mitsuokai* (Kageyama and Benno 2000) Ricci *et al*. 2025	comb. nov. [basonym: *Catenibacterium mitsuokai* Kageyama and Benno 2000]	006798	9
22	*Catenibacterium mitsuokai* subsp. *tridentinum* Ricci *et al*. 2025	subsp. nov.	006798	9
23	*Huijunlia* Wang *et al*. 2025	gen. nov.	006790	8
23	*Huijunlia imazamoxiresistens* Wang *et al*. 2025	sp. nov.	006790	8

*Homotypic synonym: ‘*Moorella carbonis*’ Böer *et al.* 2024 (effectively but not validly published name).

†Replacement name for the illegitimate family name *Moorellaceae* Lv *et al.* 2020.

‡Replacement name for the illegitimate order name *Moorellales* Lv *et al.* 2020.

§The protologue states that the type species is *I. primus* instead of *Imbroritus primus*.

The following taxonomic opinions (i.e. the emendation of circumscriptions or the creation of synonyms) do not affect the status of a name under the International Code of Nomenclature of Prokaryotes. These taxonomic opinions can neither be considered approved nor disapproved by the International Committee on Systematics of Prokaryotes.

**Table:** 

Name/authors	Article no.	Page no.
*Acetobacterium malicum* Tanaka and Pfennig 1990 emend. Spring *et al*. 2025	006783	7
*Catenibacterium mitsuokai* Kageyama and Benno 2000 emend. Ricci *et al*. 2025	006798	8
*Guptibacillus hemicentroti* (Chen *et al*. 2011) Bello *et al*. 2025 pro synon. *Guptibacillus hwajinpoensis* (Yoon *et al*. 2004) Bello *et al*. 2025^*^	006757	16
*Labrys portucalensis* Carvalho *et al*. 2008 pro synon. *Labrys neptuniae* Chou *et al*. 2007	006778	7
*Pseudalkalibacillus* Joshi *et al*. 2022 emend. Bello *et al*. 2025	006757	13
*Qipengyuania aerophila* Liu *et al*. 2022 pro synon. *Qipengyuania pacifica* Tareen *et al*. 2022	006967	17
*Streptomyces libani* Baldacci and Grein 1966 (Approved Lists 1980) pro synon. *Streptomyces nigrescens* (Sveshnikova 1957) Pridham *et al*. 1958 (Approved Lists 1980)	006711	5
*Terribacillus goriensis* (Kim *et al*. 2007) Krishnamurthi and Chakrabarti 2009 pro synon. *Terribacillus saccharophilus* An *et al*. 2007	006794	4
*Terribacillus saccharophilus* An *et al*. 2007 emend. Hauser *et al*. 2025	006794	4

*This proposal may contravene Rule 28b(1) of the International Code of Nomenclature of Prokaryotes.

## Corrigendum

In the Notification that new names of prokaryotes, new combinations and new taxonomic opinions have appeared in volume 74, part 9 of the *IJSEM* [3], the entry *Saccharococcus caldoxylosilyticus* Ahmad *et al.* 2000 pro synon. *Saccharococcus thermophilus* Nystrand 1984 must be deleted.
